# Egg Production and Biochemical Evaluation of Laying Quails Fed Diets Containing Phytase Overdosage Under Different Thermal Conditions

**DOI:** 10.3390/ani15182762

**Published:** 2025-09-22

**Authors:** Amana Fernandes Maia, Apolônio Gomes Ribeiro, Raiane dos Santos Silva, Edijanio Galdino da Silva, Luiz Arthur dos Anjos Lima, Edilson Paes Saraiva, Felisbina Luisa Pereira Guedes Queiroga, Ana Cristina Silvestre Ferreira, Xavière Rousseau, Fernando Guilherme Perazzo Costa, Ricardo Romão Guerra

**Affiliations:** 1 Departamento de Ciências Veterinárias, Universidade Federal da Paraíba, Rodovia PB-079, Areia, Paraíba 58397-000, Brazil; amanamedvet@gmail.com (A.F.M.); edijanio@veterinario.med.br (E.G.d.S.); 2 Departamento de Zootecnia, Universidade Federal da Paraíba, Rodovia PB-079, Areia, Paraíba 58397-000, Brazil; apoloniogomes962@gmail.com (A.G.R.); raianee.saantos@gmail.com (R.d.S.S.); luisarthur_@hotmail.com (L.A.d.A.L.); edilson@cca.ufpb.br (E.P.S.); 3 Centre for Animal and Veterinary Science (CECAV), University of Trás-os-Montes and Alto Douro (UTAD), 5000-801 Vila Real, Portugal; fqueirog@utad.pt (F.L.P.G.Q.); aferreir@utad.pt (A.C.S.F.); 4 AB Vista, 3 Woodstock Court, Marlborough SN8 4AN, UK; xaviere.rousseau@abvista.com

**Keywords:** *Couturnix japonica*, clinical pathology, egg production, thermal environment, phytase

## Abstract

This study evaluated the effects of increasing phytase levels on serum biochemical parameters and renal and hepatic changes in Japanese quails (*Coturnix japonica*) under different temperature conditions. A total of 720 quails were distributed in a 5 × 3 factorial design with five phytase levels (0, 500, 1000, 1500, and 3000 FTU/kg) and three temperature ranges (24, 30, and 36 °C), totaling 15 treatments with six replicates of eight birds each. Data were collected in cycles 2 and 4. The parameters measured included eggshell thickness (ST), total egg production (TEP), liver weight (LW), gamma-glutamyl transferase (GGT), alanine aminotransferase (ALT), aspartate aminotransferase (AST), alkaline phosphatase (FA), phosphorus (P), calcium (Ca), urea (URE), creatine kinase (CK), and uric acid (UA). PRO was higher at 30 °C compared to 24 °C and 36 °C. EC showed a significant interaction between phytase levels and temperature, with phytase improving EC, especially at 1500 FTU and 36 °C. PF was not affected in the second cycle but had a quadratic effect in the fourth cycle. Phytase supplementation improved shell thickness and regulated biochemical parameters, especially at high temperatures. Based on these results, a phytase level of 1500 FTU/kg is recommended to optimize performance and mitigate the negative effects of high temperatures on quail health.

## Simple Summary

Heat stress compromises the health and productivity of Japanese quails, altering biochemical parameters and affecting egg quality. This study exhibits that supplementing diets with phytase, especially at 1500 FTU/kg, helps regulate liver and kidney function as well as enhances eggshell thickness, even under high temperatures. By improving calcium and phosphorus utilization, phytase mitigates the harmful effects of heat stress, promoting better performance and welfare in laying quails and supporting sustainable poultry farming in hot climates.

## 1. Introduction

Japanese quails (*Couturnix japonica*) are domestic birds raised for egg production and meat purposes. They are considered homeothermic animals, since they have the ability to maintain a relatively constant body temperature, even in the face of environmental variations [1].

The raising of these birds has gained relevance as a low-cost and high-profit activity. This prominence is due to the modernization of management practices, the high productive potential of quails, and their low requirements in terms of space and resources. In addition, their early sexual maturity contributes accelerating economic earnings [2]. However, to ensure productivity, it is essential to consider the welfare of the birds, since inadequate conditions can negatively impact productive performance [3].

High-temperature environments pose a significant challenge to quails and lead to physiological and behavioral changes that compromise feed intake and cause damage to the intestinal epithelium. These changes result in reduced digestibility and absorption of essential nutrients from the diet [4]. Furthermore, antinutritional factors, such as phytate, aggravate the situation by reducing the bioavailability of phosphorus and interfering with the absorption of minerals, amino acids, and dietary energy [5].

Phytases represent a class of exogenous enzymes whose main function is to degrade the phytate molecule (inositol hexaphosphate—IP6) present in dietary ingredients [6]. This process releases phosphorus, calcium, and other nutrients, making them available for the animal’s body to use [7]. Calcium and phosphorus are important nutrients for bone development of animals, and supply of these minerals ensures good quality of bone structure [8]. In laying hens, such as quails, they are also essential for the formation and quality of eggshell [9].

In this regard, the aim of this study was to evaluate the effects of increasing levels of phytase on serum biochemical parameters and on renal and hepatic changes in Japanese quails subjected to different temperature conditions.

## 2. Materials and Methods

### 2.1. Ethics Committee and Experimental Site

The project received ethical approval from the Animal Care and Use Committee (CEUA) of the Federal University of Paraíba, Brazil, under protocol (process number 3695120121). This study was carried out in the climate chambers of the Bioclimatology, Behavior and Animal Welfare Research Unit, Department of Animal Science, Center for Agricultural Sciences, Federal University of Paraíba, Campus II, Areia, Paraíba.

### 2.2. Housing

The Japanese quails were housed in 3 climate chambers with an area of 19.71 m^2^. Each chamber was equipped with 30 galvanized wire cages, with dimensions of 55 × 35 × 27 cm^3^ (length × width × height), with 1 nipple drinker and one trough feeder per cage, suitable for the developmental stage of the birds.

The temperature of the chambers was controlled by means of heaters coupled with an electric thermostat and air conditioners equipped with automatic temperature control. Renewal and movement of air were carried out using axial wall exhaust fans. During each experimental test, temperature and relative humidity of air were monitored using digital maximum and minimum thermo-hygrometers in the morning and late afternoon. Temperature sensors were fixed at the level of the center-of-mass of the animals, reproducing the local microclimate. The lighting program used was 17 h/day of light throughout the experimental period.

### 2.3. Animals and Experimental Design

A total of 720 Japanese quails in the production phase were used, distributed in a completely randomized design in a 5 × 3 factorial scheme, with five phytase levels (0, 500, 1000, 1500, and 3000 FTU) and three temperature ranges (24, 30, and 36 °C), with 6 replicates with 8 birds each (48 animals per treatment), representing thermoneutral (24 °C) and thermal stress (30 and 36 °C) ranges, totaling fifteen treatments. Initially, the quails were fed a basal diet meeting their nutritional requirements. This study, however, began when the birds were eight weeks old, and they remained under the experimental treatments for 5 cycles of 21 days, totaling 105 days of the experiment. For each temperature range, 8 birds from each treatment were analyzed, totaling 120 birds. Biological material collections for this evaluation were performed during the second and fourth production cycles.

### 2.4. Experimental Diets

The diets were formulated according to the recommendations of the Brazilian Poultry and Swine Tables [10], varying only in the amount of phytase supplementation. All diets were formulated considering reductions in calcium and phosphorus levels, based on what the industrial enzyme matrix provides at 500FTU (0.165% Ca, 0.150% P) (Table 1).

During the experimental period, the birds received feed and water *ad libitum*, and the feeders were filled with the experimental feeds twice a day, at 7:00 a.m. and 4:00 p.m.

### 2.5. Data Collection

Analyses of total egg production (TEP, %), shell thickness (ST, mm), and liver weight (LW, g) were performed. The number of eggs produced was recorded throughout the experimental period. The percentage of egg production was calculated by dividing the total number of eggs per replicate by the number of birds, with corrections made for mortality when applicable.

At the end of each production cycle, four eggs per plot were selected to determine ST, which was determined using a digital Outside Micrometer (Model MDC-Lite, 293 series, Mitutoyo, Jundiaí—SP, Brazil) with an accuracy of 0.1 mm on the median line of the egg. This analysis was performed after the eggshells were dried in an oven at 55 °C.

At the end of production cycles 2 and 4, 8 birds per treatment were randomly selected and euthanized using electronarcosis to collect the digestive system organ (liver), which was weighed using analytical scales. The weights were calculated considering the weights of the birds (relative weight).

### 2.6. Collection of Biological Material

The birds were fasted for 6 h before the collection of biological material. Then, 4 mL of blood was collected from one bird per experimental plot using a puncture of the jugular vein with a 13 × 0.4 mm needle. Blood was collected in dry tubes containing a clot activator (BD Vacutainer^®^ Dry, BioScience, Hamburg, Germany) and, after 30 min of resting, the tubes were centrifuged at 3500 rpm for 1 min in a centrifuge (SL-702/RAF30, Solab, Piracicaba—SP, Brazil) Solab to separate the serum. The obtained serum was transferred to 2 mL Eppendorf tubes and stored frozen for later analysis.

### 2.7. Statistical Analysis

The data were subjected to analysis of variance (ANOVA) using the statistical software R, version 4.2.0 [11], to determine the effects of different phytase levels and temperature ranges on the measured variables. For variables showing significant differences (*p* < 0.05), Tukey’s test was conducted to compare means across different temperatures. In addition, regression analysis was employed to identify the optimal phytase level.

The variables were analyzed according to the following mathematical model:

Y_ijkl_ = μ + α_i_ + β_j_ + (αβ)_ij_ + ϵ_ijkl_,

where

- Y_ijkl_ = response variable;
- μ = overall mean;
- α_i_ = effect of the i-th level of phytase;
- β_j_ = effect of the j-th temperature range;
- (αβ)_ij_ = interaction effect between the i-th level of phytase and the j-th temperature range;
- ϵ_ijkl_ = random error term associated with each observation, assumed to be normally distributed with mean zero and constant variance.

In the case of a significant effect of phytase, the following model was applied:

Y_i_ = β_0_ + β_1_X_i_ + ϵ_i_,

where

- Y_i_ = the response variable for the i-th observation;
- β_0_ = the intercept, representing the expected value of Y when phytase is at zero (baseline level);
- X_i_ = phytase level for the i-th observation;
- β_1_ = the slope, indicating the effect of a one-unit increase in phytase on the response variable;
- ϵ_i_ = random error term for the i-th observation, assumed normally distributed with mean zero and constant variance.

## 3. Results

The mean values of shell thickness (ST), total egg production (TEP), and liver weight (LW) presented in Table 2 reveal that total egg production (TEP) was significantly influenced only by temperature (*p* < 0.0001). It was observed that the birds kept at 30 °C presented the highest percentage of egg production compared to those exposed to temperatures of 24 °C and 36 °C.

Regarding ST, a significant effect of interaction between phytase levels and temperature was observed (*p* < 0.0001). Birds not supplemented with phytase presented lower ST at high temperatures (30 °C and 36 °C), when compared to those kept at 24 °C. On the other hand, supplementation with phytase in the diet resulted in better ST, especially at higher temperatures (30 °C and 36 °C). Supplementing 1500 FTU of phytase at 36 °C provided a significant increase in ST.

Regarding LW in the second production cycle (Table 2), no significant effects of different temperatures analyzed were observed, nor was there any interaction between temperature and phytase levels. However, regression analysis revealed a quadratic effect, highlighting that the minimum estimated value occurred at 2000 FTU of enzyme (Table 5). For LW in the fourth production cycle (Table 2), a significant interaction between temperature and phytase levels was observed (*p* = 0.0095). Quails kept at 30 °C had heavier LW compared to those kept at 24 °C and 36 °C.

### Biochemical Variables

For the variables gamma-glutamyl transferase (GGT), alanine aminotransferase (ALT), aspartate aminotransferase (AST), alkaline phosphatase (AP), phosphorus (P), calcium (Ca), urea (URE), creatine kinase (CK), and uric acid (UA) related to the second production cycle (Table 3), it was observed that ALT was influenced by the interaction between temperature and phytase inclusion levels. Supplementation with phytase in the diets resulted in similar ALT levels, regardless of the temperatures analyzed. In addition, a quadratic effect was identified for the temperatures of 30 °C (*p* = 0.0161) and 36 °C (*p* = 0.0060), with maximum and minimum points estimated at 1800 FTU (Table 5), respectively.

Regarding AST, a significant effect was observed only for temperature (*p* = 0.04), with birds exposed to 30 °C presenting higher AST values than those kept at 24 °C. For AP, both temperatures and phytase levels influenced the results. Birds exposed to 36 °C presented higher AP values compared to those kept at 24 °C and 30 °C (*p* = 0.01). In addition to an increasing linear effect (*p* = 0.0404), phytase levels were increased in the diets (Table 5).

For the P and Ca variables, effects were observed only in relation to temperature. Animals kept at 24 °C presented higher P and Ca values compared to those subjected to 30 °C and 36 °C.

An interaction between temperature and phytase levels (*p* = 0.01) was also observed for URE. Phytase supplementation at different levels resulted in similar URE values across temperatures, except at the 1000 FTU level at 30 °C, where values were lower than those found at the other temperatures (24 °C and 36 °C). Regression analysis also revealed a decreasing linear effect for URE in birds kept at 24 °C as phytase levels increased (Table 5). On the other hand, birds exposed to 30 °C and 36 °C showed a quadratic effect, with a reduction in urea levels as phytase levels increased, highlighting minimum points at 1278 and 1300 FTU, respectively.

Regarding UA, there was an interaction between temperature and phytase levels (*p* = 0.01). When phytase was added to the diets, UA values were similar, except at 30 °C and 1000 FTU level, where birds had lower UA values compared to birds at the other temperatures (24 °C and 36 °C). Regression analysis revealed a decreasing linear effect on UA levels in birds kept at 36 °C (Table 5), with no significant effects for the other variables analyzed.

For the fourth production cycle (Table 4), an interaction between temperature and phytase levels (*p* = 0.02) was observed for GGT. Specifically, at 1500 FTU of phytase and 36 °C, there was a reduction in GGT levels. Regression analysis showed a quadratic effect of phytase levels at temperatures of 24 °C and 36 °C, with maximum and minimum points estimated at 1625 FTU and 1333 FTU, respectively.

An interaction effect was observed between temperature and phytase level (*p* = 0.03) for the ALT variable, at a phytase level of 3000 FTU and a temperature of 36 °C, ALT values were lower. Furthermore, regression analysis highlighted a quadratic effect of phytase levels at a temperature of 30 °C, with an estimated minimum level of 1833 FTU.

An interaction effect between temperature and phytase levels was also observed for the AP variable (*p* < 0.0001). Birds subjected to a phytase level of 1000 FTU at 24 °C presented higher AP means compared to birds at temperatures of 30 °C and 36 °C. Similarly, birds subjected to a phytase level of 1500 FTU at 30 °C presented lower AP levels compared to birds at temperatures of 24 °C and 36 °C. In the regression analysis, a quadratic effect of phytase levels at 36 °C was observed, with a maximum estimated value at 1606 FTU.

*p*-Values showed a significant interaction between temperature and phytase levels (*p* < 0.0001). Birds fed 1000 FTU of phytase at 30 °C showed higher P means than birds kept at 24 °C and 36 °C. On the other hand, birds fed 3000 FTU of phytase at 24 °C showed lower *p*-values compared to birds at the other temperatures. Regression analysis revealed a quadratic effect of phytase levels at 24 °C, with a maximum estimated point at 1625 FTU (Table 5). An increasing linear effect was also observed for phytase levels at 36 °C.

Regarding Ca, there was also a significant interaction between temperature and phytase levels (*p* < 0.0001). Birds kept at 30 °C with 0 FTU of phytase had higher Ca values than birds at 36 °C. A similar effect was observed for the 3000 FTU level of phytase. However, at 1500 FTU of phytase, birds at 30 °C had lower Ca levels than birds at 36 °C. Regression analysis revealed a quadratic effect of phytase levels at temperatures of 30 °C and 36 °C, with minimum and maximum values estimated at 1183 and 1650 FTU, respectively.

For URE, an interaction effect between temperature and phytase levels was also observed (*p* < 0.0001). Birds fed a phytase level of 500 FTUs at 30 °C had lower urea levels compared to birds at temperatures of 24 °C and 36 °C. In the regression analysis, a quadratic effect of phytase levels was observed at temperatures of 24 °C and 36 °C, with minimum values estimated at 800 and 833 FTUs, respectively. In addition, an increasing linear effect was observed for phytase levels at 30 °C, indicating that as dietary phytase levels increase, urea levels also increase at this temperature.

As to UA, no significant effects of the different temperatures analyzed were observed, nor was there any interaction between temperatures and phytase levels. However, regression analysis revealed an increasing linear effect, indicating that UA levels increased as phytase levels in the diets increased. No effects were observed for the other variables analyzed.

**Table 5:** Regression equations of the parameters for gamma glutamyl transferase (GGT); alanine aminotransferase (ALT); alkaline phosphatase (AP); phosphorus (P); calcium (Ca); urea (URE); uric acid (UA), and relative liver weight (LW) of Japanese quails fed diets containing phytase overdose in different thermal environments during production cycles 2 and 4.

Variables	Cycle	Effect	Equation	R^2^	Level
ALT (U/L)	2	Quadratic	y = −5E-07x^2^ + 0.0018x + 1.7179	0.4067	1800 FTU ^Max^
		Quadratic	y = −5E-07x^2^ − 0.0018x + 4.0212	0.998	1800 FTU ^Min^
AP (U/L)	2	Linear	y = 0.0185x + 268.75	0.2972	-
URE (mg/dL)	2	Linear	y = −0.0005x + 4.8745	0.5685	-
		Quadratic	y = 9E-07x^2^ − 0.0023x + 4.9687	0.6141	1278 FTU ^Min^
		Quadratic	y = 1E-06x^2^ − 0.0026x + 5.641	0.7541	1300 FTU ^Min^
UA (mg/dL)	2	Linear	y = −0.0003x + 3.8874	0.4993	-
GGT (U/L)	4	Quadratic	y = −4E-07x^2^ + 0.0013x + 1.6864	0.5781	1625 FTU ^Max^
		Quadratic	y = 3E-07x^2^ − 0.0008x + 2.1689	0.7938	1333 FTU ^Min^
ALT (U/L)	4	Quadratic	y = 3E-07x^2^ − 0.0011x + 2.631	0.3045	1833 FTU ^Min^
AP (U/L)	4	Quadratic	y = −0.0003x^2^ + 0.9633x + 866.88	0.5266	1606 FTU ^Max^
P (mg/dL)	4	Quadratic	y = −4E-07x^2^ + 0.0013x + 3.4947	0.6333	1625 FTU ^Max^
		Linear	y = 0.0005x + 3.8985	0.7606	-
Ca (mg/dL)	4	Quadratic	y = 3E-06x^2^ − 0.0071x + 14.294	0.9176	1183 FTU ^Min^
		Quadratic	y = −1E-06x^2^ + 0.0033x + 10.017	0.7312	1650 FTU ^Max^
URE (mg/dL)	4	Quadratic	y = 5E-07x^2^ − 0.0008x + 5.8188	0.4193	800 FTU ^Min^
		Linear	y = 0.0012x + 4.2291	0.6669	-
		Quadratic	y = 6E-07x^2^ − 0.001x + 5.6798	0.5589	833 FTU ^Min^
UA (mg/dL)	4	Linear	y = 0.0002x + 3.2809	0.4357	-
LW (%)	2	Quadratic	y = 1E-07x^2^ − 0.0004x + 2.7043	0.8768	2000 FTU ^Min^

Max = estimated maximum level; Min = estimated minimum level.

## 4. Discussion

### 4.1. Liver Weight and Egg Production Parameters

Heat stress has attracted the attention of poultry producers and researchers due to its detrimental impacts, especially on laying hens in tropical climate regions [12,13,14]. Heat stress triggers a series of physiological changes, such as oxidative stress, disturbances in acid–base balance, and weakening of the immune system [13]. These changes culminate in an increase in mortality rate and a decrease in feed efficiency, as well as in a reduction of body weight, feed intake, egg production [15], bone development [16], and eggshell thickness [12].

As highlighted by Silva et al. [17], the efficiency of calcium absorption increases proportionally to the increasing need for this mineral. Although quails are capable of withstanding temperatures of up to 30 °C, it is observed that these birds require greater amounts of vitamins and minerals in their diet due to changes in their metabolism [18]. In the context of the present study, it was found that Japanese quails kept at 30 °C are able to maintain high egg production. This highlights that, under adequate management and nutritional conditions, they can continue to be productive.

Another point observed from this study is that phytase was able to reduce the effects of heat stress. Birds presented greater ST at high temperatures, especially when supplemented with 1500 FTU. The thickest shell result was at a temperature of 36 °C, which is considered a severe stress condition. These findings demonstrate the effectiveness of the enzyme in promoting the release of nutrients to the animals. Furthermore, this study confirms that at temperatures of 30 °C, where the demand for calcium is greater compared to the thermoneutral temperature (24 °C), animals demonstrated greater efficiency in the use of calcium from the diet, justified by greater egg production at this temperature.

The improvement in mineral digestibility in animals supplemented with phytase can also be observed in liver mass. The liver, as the main organ involved in the metabolism of birds, is sensitive to nutritional changes. In this study, it was observed that by increasing levels of phytase in the diet, liver weight reduced, up to a level of 2000 FTU of enzyme. This can be explained by the fact that the enzyme degrades the phytate molecule present in ingredients of the diets, increasing the availability of essential nutrients for the birds. This action not only improves the efficiency of the absorption of essential nutrients for the birds but can also positively impact the overall health of the liver, resulting in better productive performance in the birds [14].

Quails kept at high temperatures have higher nutritional requirements compared to those kept under thermal comfort conditions [12]. This increased demand for nutrients is related to reduced feed intake, a response to thermal stress. However, in this study, even under moderately stressful environment (30 °C), the birds demonstrated efficient use of dietary nutrients. This efficiency is due to the action of phytase, which promotes the breakdown of phytate, resulting in better metabolization of nutrients and, consequently, an increase in liver weight.

According to Rodrigues et al. [19], quails raised under 12-h heat cycles (32 °C) and at thermoneutral temperatures (24 °C) did not show changes in liver weight. However, in our study, conducted at temperatures of 24, 30, and 36 °C, an increase in liver weight was observed in birds subjected to 30 °C during the fourth production cycle. This increase can be attributed to phytase supplementation, which helped mitigate the effects of heat stress and allowed for a more efficient metabolization of dietary nutrients.

### 4.2. Serum Biochemistry

#### 4.2.1. Second Production Cycle

Gamma-glutamyl transferase (GGT) is a membrane enzyme present in various tissues, but in birds, it is mainly associated with the biliary and renal epithelia. Elevated levels of GGT in the blood of birds may be indicative of active liver injury. In the present research, serum levels for GGT during the second and fourth production cycles remained within the normal standards for birds (0–10 U/L) [20,21], indicating a normal state of the birds during this period.

The normal state of the liver can also be assessed using the concentration of alanine aminotransferase (ALT) in blood, since high levels of this enzyme may indicate liver problems. In this study, it was observed that the greater availability of essential minerals and amino acids, provided by the action of phytase enzyme in the degradation of phytate molecule, may have contributed to the reduction of metabolic stress on the liver, reflected in lower levels of ALT in blood. It was observed that, at temperatures of 24 °C and 30 °C, ALT levels were lower in the basal diet without phytase supplementation (0 FTU), suggesting that birds may have presented lower metabolic stress under these thermal conditions. However, at 36 °C, a temperature characterized as more stressful, the absence of phytase supplementation resulted in a significant increase in ALT levels. These results suggest that under conditions of high thermal stress, the presence of phytase may be crucial to maintain hepatic integrity in birds. ALT values in this present research remained below the normal reference values for birds, which are 5–11 U/L, for non-carnivorous and non-migratory birds [22]. However, in several bird species, ALT may present normal activity levels below the sensitivity threshold of some analyzers [23]. Therefore, low ALT activity in birds’ bodies may be of a physiological nature.

Aspartate aminotransferase (AST) is a widely distributed enzyme in birds. It is found in high concentrations in various organs and tissues, including the heart, liver, skeletal muscles, kidneys, and brain. However, its distribution varies among avian species, which prevents it from being considered a liver-specific enzyme, also due to its high sensitivity to muscle tissue [24]. Although blood AST activity may be a sensitive marker for hepatocellular disorders in birds, it is not specific for this purpose [25]. Therefore, to differentiate between liver and muscle damage, it is essential to measure AST in conjunction with a muscle-specific enzyme, such as creatine kinase (CK). Although these findings revealed above normal AST values (<275 IU/L) [26] for animals subjected to a temperature of 30 °C, results for CK remained similar among animals across different temperatures, and thus the elevation of AST in birds in this study can be ruled out to be of muscular origin.

Alkaline phosphatase (AP) is a fundamental enzyme for calcium and phosphorus metabolism in birds. It plays an important role in chondrogenic (cartilage formation) and osteoblastic (bone formation) activities, which are essential for adequate growth of these species. AP has low activity in the liver of birds, and its increase in serum or blood plasma is strongly associated with osteoblastic activity and several bone alterations, such as accelerated growth, fracture healing, osteomyelitis, secondary nutritional hyperparathyroidism, and neoplasia [24].

Under heat stress, bone metabolism in Japanese quails can be significantly affected, resulting in elevations in alkaline phosphatase (AP) levels. This increase can be attributed to two main factors: increased osteoblastic activity, which is responsible for the formation of new bone tissue, and the release of AP by bone cells in response to stress [12]. Elevated osteoblastic activity may occur as an attempt by the organism to compensate for the mobilization of essential calcium and phosphorus for eggshell formation, which may be compromised under extreme heat conditions [27].

The inclusion of phytase in diets can significantly improve phosphorus bioavailability, since the enzyme breaks down phytic acid present in plant ingredients, releasing inorganic phosphorus that can be absorbed and used by the body. This increase in phosphorus availability stimulates bone metabolism, promoting adequate bone mineralization and consequently increasing the activity of enzymes related to this process. such as alkaline phosphatase. The increasing linear effect in alkaline phosphatase levels with increasing phytase levels observed in our study can be attributed to the greater release of phosphorus by the action of the enzyme, which improves the availability of this essential mineral for bone metabolism, favoring the development and maintenance of skeletal health in birds.

For laying quails, calcium (Ca) and phosphorus (P) minerals are essential, and their availability is most crucial during the laying period. Among all minerals, calcium and phosphorus play the most fundamental role in the construction of the skeleton, constituting 80 to 85% of its structure. They are essential for the formation of eggshells and muscle development. Thus, these minerals are indispensable for the proper functioning of the animal’s body [12].

In our study, serum Ca and P levels of birds kept at temperatures of 30 and 36 °C were slightly below the recommendations (Ca: 20 to 30 mg/dL and P: 5 to 7 g/L) [26]. This may be explained by the fact that in high-temperature environments, birds tend to reduce feed intake, thus limiting the availability of calcium and phosphorus in the diet. This decrease in intake may affect variations in the absorption and mobilization of these minerals in the blood, since calcium and phosphorus are essential for several physiological functions, including eggshell formation and maintenance of bone health.

When evaluating the effects of acute and chronic heat stress on performance, egg quality, body temperature, and blood gas parameters in laying hens, Barrett et al. [28] observed that under heat stress, birds tend to have reduced serum Ca levels. Similar results were found in studies with laying hens subjected to heat stress, which demonstrated a decrease in plasma P concentrations in animals exposed to temperatures of 34 °C, indicating the occurrence of severe heat stress [29,30,31,32].

Furthermore, Yahav et al. [33] suggest that the reduction in blood ion concentrations may be related to an increase in blood plasma volume during heat stress. This increase occurs to reduce resistance to blood flow, facilitating more efficient dissipation of heat.

Urea is a product of protein catabolism, synthesized in the liver and excreted mainly by glomerular filtration in the kidneys. Its concentration in the blood is influenced by several factors, including diet—especially protein intake, hydration status of the bird, rate of renal excretion, and liver function [34]. Unlike uric acid, whose excretion is less affected by hydration, the concentration of urea in the blood is significantly influenced by the hydration status of the birds. The elevation of URE values in the present study may represent a state of dehydration of the animal, since most of the URE concentration values that were above the reference values (<5 mg/dL for non-carnivorous birds) [26] are present in an environment of heat discomfort, which may justify these values, since UA concentrations are all within the normal range, thus rules out the possibility of kidney injury.

Increasing the levels of phytase in diets reduced serum URE levels in birds, especially in warmer environments. This occurred because the enzyme promoted greater release of nutrients, energy, and antioxidant factors, and also improved blood pH stability, increasing mineral availability. This effect contributes to a possible acid–base balance of blood and to the regulation of electrolytes such as potassium, sodium, and calcium [12]. As a result, birds tend to use less rapid and shallow breathing (panting) to promote evaporative heat loss, which helps to maintain a stable body hydration state.

Like urea, blood uric acid concentrations are slightly affected by the hydration status of birds but reflect the functional capacity of the proximal renal tubules. Therefore, measuring uric acid levels is one of the main laboratory parameters used to identify possible kidney diseases in birds. As mentioned previously, serum uric acid levels in the birds in the present study are within the normal range (<15 mg/dL) [26,35], indicating normal renal function.

#### 4.2.2. Fourth Production Cycle

When used in poultry feed, phytase can reduce the effects of heat stress, since it not only enables the degradation of the phytate molecule present in diet ingredients, releasing phosphorus and calcium [7], which are important for the body and bone development of animals [36], but also releases minerals and vitamins in the process, such as zinc and vitamin D, which are linked to bone development [16,37] and also act as antioxidant factors, removing free radicals and protecting cell membranes from oxidative stress caused by high temperatures [12].

In the present study, the inclusion of phytase positively influenced the biochemical parameters of the quails. It was observed that gamma-glutamyl transferase (GGT) levels remained similar regardless of the different temperatures tested (24 °C, 30 °C, and 36 °C). Furthermore, aspartate aminotransferase (AST) levels were within the established reference values for female quails (<402 U/L) [20], suggesting that there was no significant liver or muscle damage. Although alanine aminotransferase (ALT) levels were slightly below the reference value, this may indicate good liver health since reduced ALT levels suggest the absence of stress or liver injury in the birds studied.

Increasing addition of phytase in the birds’ diet resulted in similar levels of alkaline phosphatase, phosphorus, and serum calcium. regardless of temperature variations (24 °C, 30 °C, and 36 °C). This stability in biochemical parameters is essential for the bone and mineral metabolism of quails, ensuring adequate availability and utilization of phosphorus and calcium, which are essential elements not only for skeletal development but also for egg production.

Phytase not only improves phosphorus bioavailability but can also influence protein digestibility, resulting in better nutrient utilization and reduced excretion of nitrogen compounds. These dynamics are crucial to optimizing bird nutrition and minimizing the environmental impacts of poultry production. Serum urea levels during the fourth production cycle in our study were slightly above reference values, which may be related to the greater mobilization of proteins to meet the nutritional demands of birds, especially under hot environments.

Lelis et al. [38] evaluated dietary supplementation of phytase in broilers using doses of 250 and 500 FTU/kg of feed. The results showed that this enzyme significantly increased the digestibility coefficients of crude protein and phosphorus, with the best results obtained at the dose of 500 FTU/kg. This highlights the positive influence of phytase in improving digestibility and increasing the availability of nutrients for the birds.

Additionally, diets containing phytase may lead to a temporary increase in blood urea nitrogen (urea) levels in birds. This occurs because the body produces ureic nitrogen by breaking down proteins released due to the enzyme’s action on phytate. However, this increase in urea levels is not a cause for concern, since uric acid levels remain within the recommended range for the production phase. This suggests that, although there is a small increase in serum urea levels, the overall health of the birds has not been compromised.

Creatine kinase (CK) is an enzyme that produces and utilizes phosphocreatine to transfer energy to maintain tissue and cellular energy homeostasis and is considered a primary controller of cellular energy homeostasis. Its activity in plasma/serum has been commonly used to assess tissue damage, since CK is released into the bloodstream during the damage [39]. Although these findings for the fourth production cycle indicate higher CK values than the reference values (100–500 U/L) [26,35], studies indicate that as animals increase in age, their serum CK levels may increase [40]. This may be related to birds’ muscular development. It is also worth mentioning that some studies have already shown that variations in the activity of these enzymes occur due to physiological variations [41]. Another factor to consider is that in birds of prey, intense muscular activity during restraint for blood collection can significantly increase CK levels due to associated stress [26].

## 5. Conclusions

This study demonstrated that phytase supplementation improved eggshell thickness and positively influenced serum biochemical parameters of quails, including reduced ALT levels under heat stress and stabilization of serum calcium, phosphorus, and alkaline phosphatase. These effects were particularly evident at 36 °C. A temperature of 30 °C was the most favorable for egg production, while phytase effectively mitigated the negative impacts of heat stress. Based on these results, a phytase level of 1500 FTU/kg is recommended to optimize performance and maintain biochemical stability in quails under high-temperature conditions.

## Figures and Tables

**Table 1:** Experimental diets containing five levels of phytase (0, 500, 1000, 1500, and 3000 FTU) and reductions in phosphorus and calcium levels based on the 500 FTU matrix of the enzyme for Japanese quails in the production phase.

Treatments		T1	T2	T3	T4	T5
Ingredients	Unit	0 FTU	500 FTU	1000 FTU	1500 FTU	3000 FTU
Corn-7.88%	g/kg	597	597	597	597	597
Soybean meal 45.22%	g/kg	305	305	305	305	305
Soybean oil	g/kg	6.67	6.67	6.67	6.67	6.67
DL-methionine	g/kg	3.98	3.98	3.98	3.98	3.98
L-Lysine HCl	g/kg	2.65	2.65	2.65	2.65	2.65
L-threonine	g/kg	0.35	0.35	0.35	0.35	0.35
Limestone	g/kg	74.37	74.37	74.37	74.37	74.37
Dicalcium phosphate 18.5%	g/kg	4.00	4.00	4.00	4.00	4.00
Common salt	g/kg	3.45	3.45	3.45	3.45	3.45
Mineral premix ^a^	g/kg	0.50	0.50	0.50	0.50	0.50
Vitamin premix ^b^	g/kg	0.25	0.25	0.25	0.25	0.25
Choline	g/kg	0.70	0.70	0.70	0.70	0.70
Antioxidante	g/kg	0.10	0.10	0.10	0.10	0.10
Inert ^c^	g/kg	0.60	0.50	0.40	0.30	0.00
Phytase ^d^	g/kg	0.00	0.10	0.20	0.30	0.60
Total		1000	1000	1000	1000	1000
Nutrients	Unit					
Phytase	FTU/kg	0	500	1000	1500	3000
Metabolizable energy	kcal/kg	2800	2800	2800	2800	2800
Crude protein	g/kg	190.00	190.00	190.00	190.00	190.00
Calcium	g/kg	29.93	29.93	29.93	29.93	29.93
Phosphorus total	g/kg	3.94	3.94	3.94	3.94	3.94
Available phosphorus	g/kg	1.77	1.77	1.77	1.77	1.77
Potassium	g/kg	7.32	7.32	7.32	7.32	7.32
Sodium	g/kg	1.55	1.55	1.55	1.55	1.55
Chlorine	g/kg	3.19	3.19	3.19	3.19	3.19
Mogin number	mEq/kg	164.59	164.59	164.59	164.59	164.59
Digestible amino acid (%)						
Digestible methionine	g/kg	6.47	6.47	6.47	6.47	6.47
Digestible methi. + cystine	g/kg	9.08	9.08	9.08	9.08	9.08
Digestible lysine	g/kg	11.07	11.07	11.07	11.07	11.07
Digestible threonine	g/kg	6.75	6.75	6.75	6.75	6.75
Digestible tryptophan	g/kg	2.07	2.07	2.07	2.07	2.07
Digestible valine	g/kg	7.98	7.98	7.98	7.98	7.98

The treatments described above were subjected to three different temperatures (24, 30, and 36 °C). ^a^ Mineral premix (concentration/kg of product): Mn—60 g, Fe—80 g, Zn—50 g, Cu—10 g, Co—2 g, I—1 g, and Se—250 mg. ^b^ Vitamin premix (concentration/kg of product): Vit. A—15 mil UI, Vit. D3—1,500,000 UI, Vit. E—wm 15,000, Vit.B1—2.0 g, Vit. B2—4.0 g, Vit. B6—3.0 g, Vit. B12—0015 g, nicotinic acid—25 g, pantothenic acid—10 g, Vit. K3—3.0 g, and folic acid—1.0 g. ^c^ Inert = Kaolin. ^d^ Phytase enzyme = 100 g/ton provides 500 FTUs/kg of feed.

**Table 2:** Average values of shell thickness, total egg production, and liver weight related to the second and fourth laying cycles of Japanese quails fed varying phytase doses and subjected to three different temperatures.

Parameters	Temperature	Phytase (FTU/Kg)					Mean	CV%	*p*-Value			
		0	500	1000	1500	3000						
									Temperatury	Phytase	Temp. × Phy.	Regression
ST (mm)	24	0.427 ^A^	0.406 ^A^	0.410 ^A^	0.404 ^B^	0.406 ^A^	0.411					
	30	0.402 ^B^	0.395 ^A^	0.411 ^A^	0.401 ^B^	0.407 ^A^	0.403	3.602	0.1180	0.0329	<0.0001	NS
	36	0.387 ^B^	0.398 ^A^	0.394 ^A^	0.443 ^A^	0.412 ^A^	0.407					
	Mean	0.405	0.399	0.405	0.415	0.408						
TEP (%)	24	66.013	66.749	68.071	70.074	65.365	67.254 ^B^					
	30	80.549	76.297	82.280	80.740	77.122	79.398 ^A^	11.670	<0.0001	0.7607	0.9266	NS
	36	66.379	64.943	64.313	61.510	62.570	63.943 ^B^					
	Mean	70.980	69.330	71.555	70.775	68.352						
LW (%) Second cycle	24	2.567	2.514	2.297	2.714	2.523	2.523					
	30	2.710	2.618	2.459	2.447	2.751	2.597	14.297	0.5053	0.0291	0.0654	0.0037 **
	36	2.769	2.714	2.441	2.111	2.515	2.510					
	Mean	2.682	2.615	2.399	2.424	2.596						
LW (%) Fourth cycle	24	2.767 ^AB^	2.008 ^B^	2.411 ^A^	2.429 ^A^	2.653 ^AB^	2.454					
	30	3.013 ^A^	2.814 ^A^	2.377 ^A^	2.327 ^A^	3.017 ^A^	2.710	7.500	<0.0001	0.3383	0.0095	NS
	36	2.149 ^B^	2.431 ^AB^	2.192 ^A^	2.166 ^A^	2.112 ^B^	2.210					
	Mean	2.643	2.418	2.192	2.308	2.594						

CV% = coefficient of variation; NS = not significant; ST = shell thickness; TEP = total egg production; LW = liver weight. Averages followed by different letters within columns compare the temperatures between each phytase concentration, using the Tukey test (*p* < 0.05). ** Quadratic Effect.

**Table 3:** Gamma-glutamyl transferase, alanine aminotransferase, aspartate aminotransferase, alkaline phosphatase, phosphorus, calcium, urea, creatine kinase, and uric acid of Japanese quails fed diets containing increasing levels of phytase and subjected to different temperatures during the second production cycle.

Parameters	Temperature	Phytase (FTU/Kg)					Mean	CV%	*p*-Value			
		0	500	1000	1500	3000						
									Temperatury	Phytase	Temp. × Phy.	Regression
GGT (U/L)	24	0.37	1.38	3.34	5.22	0.58	2.18					
	30	0.67	4.12	1.10	0.37	0.66	1.38	25.89	0.84	0.28	0.60	NS
	36	0.67	7.30	1.10	0.52	0.41	1.99					
	Mean	0.57	4.27	1.85	2.03	0.55	0.57					
ALT (U/L)	24	2.20 ^B^	3.85 ^A^	3.48 ^A^	2.70 ^A^	2.40 ^A^	2.92					NS
	30	1.10 ^B^	3.68 ^A^	2.82 ^A^	2.74 ^A^	2.43 ^A^	2.55	26.79	0.06	0.03	0.01	0.0161 **
	36	4.03 ^A^	3.23 ^A^	2.80 ^A^	2.51 ^A^	3.39 ^A^	3.19					0.0060 **
	Mean	2.44	3.58	3.03	2.65	2.74						
AST (U/L)	24	264.9	218.0	272.2	238.4	247.9	248.3 ^B^					
	30	243.7	277.0	307.2	302.9	289.8	284.1 ^A^	20.89	0.04	0.86	0.11	NS
	36	278.5	303.9	223.5	306.9	278.2	278.2 ^AB^					
	Mean	262.4	266.3	267.6	282.8	271.0						
AP (U/L)	24	260.6	303.8	262.7	260.0	303.9	278.2 ^B^					
	30	199.1	300.5	212.1	223.8	321.7	251.5 ^B^	27.98	0.01	0.01	0.91	0.041 *
	36	284.6	392.6	314.5	354.9	369.8	343.3 ^A^					
	Mean	248.1	332.3	263.1	279.6	331.8						
P (mg/dL)	24	5.20	5.05	5.22	5.71	5.29	5.29 ^A^					
	30	4.61	4.54	4.38	4.16	4.84	4.50 ^B^	19.38	0.01	0.82	0.83	NS
	36	4.92	4.47	3.91	4.22	4.20	4.34 ^B^					
	Mean	4.91	4.68	4.50	4.70	4.77						
Ca (mg/dL)	24	19.18	18.12	18.50	21.45	19.88	19.42 ^A^					
	30	18.73	16.05	17.62	19.01	16.34	17.55 ^B^	12.00	0.02	0.19	0.17	NS
	36	17.28	16.68	16.92	15.65	17.40	16.78 ^B^					
	Mean	18.40	16.95	17.68	18.70	17.87						
URE (mg/dL)	24	4.36 ^A^	5.44 ^A^	4.38 ^A^	3.70 ^A^	3.35 ^B^	4.25					0.006 *
	30	4.46 ^A^	5.28 ^A^	2.65 ^B^	3.49 ^A^	5.83 ^AB^	4.34	17.39	0.02	<0.0001	0.01	<0.001 **
	36	5.00 ^A^	5.83 ^A^	3.80 ^A^	3.55 ^A^	7.25 ^A^	5.09					<0.001 **
	Mean	4.60	5.52	3.61	3.58	5.48						
CK (U/L)	24	770.7	564.4	748.6	539.4	660.5	656.7					
	30	741.6	740.2	748.3	485.1	674.2	677.9	26.61	0.94	0.05	0.37	NS
	36	521.2	683.2	755.7	567.6	799.6	665.5					
	Mean	677.8	662.6	750.9	530.7	711.4						
UA (mg/dL)	24	3.49 ^A^	2.78 ^A^	3.99 ^A^	3.87 ^A^	3.24 ^A^	3.47					NS
	30	3.28 ^A^	2.75 ^A^	2.41 ^B^	3.14 ^A^	3.25 ^A^	2.97	20.14	0.02	0.15	0.01	NS
	36	4.04 ^A^	3.60 ^A^	3.93 ^A^	3.01 ^A^	3.23 ^A^	3.56					0.051 *
	Mean	3.60	3.04	3.44	3.34	3.24						

CV% = coefficient of variation; NS = not significant. Gamma-glutamyl transferase (GGT); alanine aminotransferase (ALT); aspartate aminotransferase (AST); alkaline phosphatase (AP); phosphorus (P); calcium (Ca); urea (URE); creatine kinase (CK); and uric acid (UA). Averages followed by different letters within columns compare the temperatures at each phytase concentration, using the Tukey test (*p* < 0.05). * Linear effect; ** quadratic effect.

**Table 4:** Gamma-glutamyl transferase, alanine aminotransferase, aspartate aminotransferase, alkaline phosphatase, phosphorus, calcium, urea, creatine kinase, and uric acid of Japanese quails fed diets containing phytase overdose and subjected to heat stress during the fourth laying cycle.

Parameters	Temperature	Phytase (FTU/Kg)					Mean	CV%	*p*-Value			
		0	500	1000	1500	3000						
									Temperatury	Phytase	Temp. × Phy.	Regression
GGT (U/L)	24	1.67 ^A^	2.50 ^A^	2.00 ^A^	3.10 ^A^	1.90 ^A^	2.23					0.013 **
	30	2.08 ^A^	2.20 ^A^	1.75 ^A^	2.18 ^A^	1.30 ^A^	1.90	23.87	0.18	0.04	0.02	NS
	36	2.10 ^A^	2.08 ^A^	1.43 ^A^	1.27 ^B^	2.50 ^A^	1.97					0.009 **
	Mean	1.95	2.26	1.72	2.35	1.90						
ALT (U/L)	24	1.88 ^A^	1.48 ^A^	1.50 ^A^	1.55 ^A^	2.05 ^A^	1.69					NS
	30	2.20 ^A^	3.10 ^A^	1.54 ^A^	1.60 ^A^	2.55 ^A^	2.20	22.87	0.04	0.32	0.03	0.025 **
	36	1.93 ^A^	1.82 ^A^	1.76 ^A^	2.30 ^A^	1.13 ^B^	1.79					NS
	Mean	2.00	2.13	1.60	1.82	1.91						
AST (U/L)	24	273.7	258.8	304.0	267.2	322.0	285.1					
	30	267.6	276.6	304.3	294.4	294.7	287.5	19.52	0.92	0.40	0.95	NS
	36	269.8	300.5	304.5	286.9	296.1	291.6					
	Mean	270.3	278.6	304.3	282.8	304.3						
AP (U/L)	24	1844.3 ^A^	822.2 ^A^	2032.6 ^A^	1942.4 ^A^	755.6 ^A^	1479.4					NS
	30	656.0 ^A^	1373.5 ^A^	646.7 ^B^	611.9 ^B^	1622.0 ^A^	982.0	27.24	0.03	0.31	<0.001	NS
	36	757.9 ^A^	1676.0 ^A^	940.4 ^B^	1788.2 ^A^	632.4 ^A^	1159.0					0.008 **
	Mean	1086.1	1290.6	1206.6	1447.5	1003.3						
P (mg/dL)	24	3.65 ^A^	3.96 ^A^	3.99 ^B^	5.06 ^A^	3.90 ^B^	4.110					0.004 **
	30	4.81 ^A^	3.43 ^A^	4.87 ^A^	4.13 ^A^	4.53 ^AB^	4.354	13.55	0.26	0.03	<0.001	NS
	36	4.15 ^A^	3.78 ^A^	4.17 ^B^	5.02 ^A^	5.30 ^A^	4.366					<0.001 *
	Mean	4.205	3.724	4.145	4.737	4.574						
Ca (mg/dL)	24	10.83 ^AB^	9.59 ^A^	12.41 ^A^	11.49 ^AB^	10.50 ^B^	10.96					NS
	30	13.41 ^A^	12.97 ^A^	10.04 ^A^	8.87 ^B^	17.98 ^A^	12.65	19.23	0.03	0.08	<0.001	<0.001 **
	36	10.27 ^B^	11.32 ^A^	11.40 ^A^	13.61 ^A^	11.03 ^B^	11.52					0.035 **
	Mean	11.50	11.29	11.28	11.32	13.17						
URE (mg/dL)	24	5.67 ^A^	6.63 ^A^	3.55 ^A^	7.00 ^A^	8.01 ^A^	6.17					0.002 **
	30	5.26 ^A^	3.44 ^B^	5.03 ^A^	6.64 ^A^	7.69 ^A^	5.61	13.90	0.04	<0.001	<0.001	<0.001 *
	36	5.58^A^	6.21^A^	3.57 ^A^	6.63 ^A^	8.14 ^A^	6.02					0.005 **
	Mean	5.50	5.42	4.05	6.76	7.94						
CK (U/L)	24	443.1 ^A^	619.3 ^A^	482.4 ^A^	509.5 ^B^	421.1 ^B^	495.1					NS
	30	701.4 ^A^	510.0 ^A^	400.7 ^A^	802.8 ^A^	714.6 ^A^	625.9	24.34	0.02	0.22	0.01	NS
	36	524.1 ^A^	644.4 ^A^	585.9 ^A^	533.1 ^B^	577.0 ^AB^	572.9					NS
	Mean	556.2	591.2	489.7	615.1	570.9						
UA (mg/dL)	24	2.93	3.85	3.52	2.96	3.70	3.39					
	30	3.01	3.66	3.29	3.48	3.85	3.46	17.12	0.44	0.01	0.23	0.015 *
	36	3.77	3.38	3.46	3.20	4.19	3.60					
	Mean	3.24	3.63	3.42	3.21	3.91						

CV% = coefficient of variation; NS = not significant. Gamma-glutamyl transferase (GGT); alanine aminotransferase (ALT); aspartate aminotransferase (AST); alkaline phosphatase (AP); phosphorus (P); calcium (Ca); urea (URE); creatine kinase (CK); and uric acid (UA). Averages followed by different letters within columns compare the temperatures within each phytase concentration, using the Tukey test (*p* < 0.05). * Linear effect; ** quadratic effect.

## Data Availability

The data supporting the findings of this study are available upon request from the corresponding author.

## References

[1] Güngören A., Güngören G., Şimşek U.G., Yilmaz O., Bahşi M., Aslan S. (2023). Quality properties and fatty acids composition of breast meat from Japanese quails with different varieties grown under warm climate. Veterinaria.

[2] Santos T.C., Gates R.S., Tinôco I.F.F., Zolnier S., Baêta F.C. (2017). Behavior of Japanese quail in different air velocities and air temperatures. Pesqui. Agropecu. Bras..

[3] Santos T.C., Gates R.S., Tinôco I.F.F., Zolnier S., Rocha K.S.O., Freitas L.C.S.R. (2019). Productive performance and surface temperatures of Japanese quail exposed to different environment conditions at start of lay. Poult. Sci..

[4] Rostagno M.H. (2020). Effects of heat stress on the gut health of poultry. J. Anim. Sci..

[5] Bernardes R.D., Oliveira C.H., Calderano A.A., Ferreira R.S., Dias K.M.M., Almeida B.F., Aleixo P.E., Albino L.F.T. (2022). Effect of phytase and protease combination on performance. metabolizable energy. and amino acid digestibility of broilers fed nutrient-restricted diets. Rev. Bras. Zootec..

[6] Bello A., Dersjant-Li Y., Korver D.R. (2019). The efficacy of 2 phytases on inositol phosphate degradation in different segments of the gastrointestinal tract. calcium and phosphorus digestibility. and bone quality of broilers. Poult. Sci..

[7] Hirvonen J., Liljavirta J., Saarinen M.T., Lehtinen M.J., Ahonen I., Nurminen P.I. (2019). Effect of Phytase on in Vitro Hydrolysis of Phytate and the Formation of myo-Inositol Phosphate Esters in Various Feed Materials. J. Agric. Food Chem..

[8] Wawrzyniak A., Balawender K. (2022). Structural and Metabolic Changes in Bone. Animals.

[9] Dijkslag M.A., Kwakkel R.P., Martin-Chaves E., Alfonso-Carrillo C., Walvoort C., Navarro-Villa A. (2021). The effects of dietary calcium and phosphorus level. and feed form during rearing on growth performance. bone traits and egg production in brown egg-type pullets from 0 to 32 weeks of age. Poult. Sci..

[10] Rostagno H.S., Albino L.F.T., Hannas M.I., Donzele J.L., Sakomura N.K., Perazzo F.G., Saraiva A., Teixeira M.L., Rodrigues P.B., Oliveira R.F. (2017). Tabelas Brasileiras Para Aves e Suínos: Composição de Alimentos e Exigências Nutricionais.

[11] R Core Team (2022). R: A Language and Environment for Statistical Computing, Version. 4.2.0..

[12] Ribeiro A.G., Silva R.S., Costa F.S., Silva E.G., Santos Ribeiro J.E., Saraiva E.P., Costa F.G.P., Guerra R.R. (2024). Phytase super-dosing modulates bone parameters and the concentration of the calcium epithelial carrier Calbindin-D28k in quails (*Coturnix japonica*) under thermal stress. Anim. Prod. Sci..

[13] Ribeiro A.G., Silva R.S., Silva D.A., Nascimento J.C.S., Souza L.F.A., Silva E.G., Ribeiro J.E.S., Campos D.B., Alves C.V.B.V., Saraiva E.P. (2024). Heat stress in Japanese quails (*Coturnix japonica*): Benefits of phytase supplementation. Animals.

[14] Ribeiro A.G., Silva R.S., Alves C.V.B.V., Campos D.B., Silva D.A., Nascimento J.C.S., Silva E.G., Saraiva E.P., Costa F.G.P., Pereira W.E. (2025). Gene expression of calcium transporters Calbindin-D28K and TRPV6 in Japanese quails (*Coturnix japonica*) subjected to phytase super-dosing and under different temperatures. Poult. Sci..

[15] Wasti S., Sah N., Mishra B. (2020). Impact of heat stress on poultry health and performances. and potential mitigation strategies. Animals.

[16] Zhang H., Majdeddin M., Gaublomme D., Taminiau B., Boone M., Elewaut D., Daube G., Josipovic I., Zhang K., Michiels J. (2021). 25hydroxycholecalciferol reverses heat induced alterations in bone quality in finisher broilers associated with effects on intestinal integrity and inflammation. J. Anim. Sci. Biotechnol..

[17] Silva R.M., Furlan A.C., Ton A.P.S., Martins E.N., Scherer C., Murakami A.E. (2009). Calcium and phosphorus requirements of finishing meat quail. Rev. Bras. Zootec..

[18] Truong L., Miller M.R., Sainz RDKing A.J. (2023). Changes in Japanese quail (Coturnix coturnix japonica) blood gases and electrolytes in response to multigenerational heat stress. PlosClimate.

[19] Rodrigues V.P., Furtado D.A., Ribeiro N.L., Rodrigues L.R., Abreu C.G., Sousa J.G. (2022). Magnesium in the water of japanese quails kept under comfort zone and under thermal stress. Semin. Cienc. Agrar..

[20] Scholtz N., Halle I., Flachowsky G., Sauerwein H. (2009). Serum chemistry reference values in adult Japanese quail (*Coturnix coturnix japonica*) including sex-related differences. Poult. Sci..

[21] Rezende M.S. (2017). Blood Biochemical Profile of a Heavy Line of Broiler Chickens. Ph.D. Thesis.

[22] Carpenter J.W., Marion C.J. (2018). Exotic Animal Formulary.

[23] Hochleithner M., Ritchie B.W., Harrison G.J., Harrison L.R. (1994). Biochemistries. Avian Medicine: Principles and Application.

[24] Barbosa T.S., Mori C.K., Polônio L.B., Ponsano E.H.G., Ciarlini P.C. (2011). Serum biochemical profile of laying hens in the region of Araçatuba. SP. Semin. Cienc. Agrar..

[25] Grunkemeyer V.L. (2010). Advanced Diagnostic Approaches and Current Management of Avian Hepatic Disorders. Vet. Clin. N. Am. Exot. Anim. Pract..

[26] Thrall M.A., Baker D.C., Campbell T.W., Denicola D.B., Fettman M.J., Lassen E.D., Rebar A., Weiser G. (2004). Veterinary Hematology and Clinical Chemistry.

[27] Franzini B.D., Cruz L.C.F., Sampaio S.A., Borges K.F., Barros H.S.S., Santana F.X.C., Gouveia A.B.V.S., Paulo L.M., Minafra C.S. (2022). Blood hematological and hormonal indicators of stress in poultry. Research. Soc. Dev..

[28] Barrett N.W., Rowland K., Schmidt C.J., Lamont S.J., Rothschild M.F., Ashwell C.M., Persia M.E. (2019). Effects of acute and chronic heat stress on the performance. egg quality. body temperature. and blood gas parameters of laying hens. Poult. Sci..

[29] Wolfenson D., Sklan D., Graber Y., Kedar O., Bengal I., Hurwitz S. (1987). Absorption of protein. fatty acids and minerals in young turkeys under heat and cold stress. Br. Poult. Sci..

[30] Ait-Boulahsen A., Garlich J.D., Edens F.W. (1993). Calcium deficiency and food deprivation improve the response of chickens to acute heat stress. J. Nutr..

[31] Usayran N., Farran M.T., Awadallah H.H., Al-Hawi I.R., Asmar R.J., Ashkarian V.M. (2001). Effects of Added Dietary Fat and Phosphorus on the Performance and Egg Quality of Laying Hens Subjected to a Constant High Environmental Temperature. Poult. Sci..

[32] Persia M.E., Utterback P.L., Biggs P.E., Koelkebeck K.W., Parsons C.M. (2003). Interrelationship between environmental temperature and dietary nonphytate phosphorus in laying hens. Poult. Sci..

[33] Yahav S., Straschnow A., Plavnik I., Hurwitz S. (1997). Blood system response of chickens to changes in environmental temperature. Poult. Sci..

[34] Bernardino M.G.S. (2016). Avaliação dos Parâmetros Hematológicos e Bioquímicos de Codornas Japonesas (Coturnix Coturnix Japonica) em Diferentes Faixas Etárias. Dissertação (Mestre em Ciência Animal). Programa de Pós-Graduação em Ciência Animal do Centro de Ciências Agrárias da Universidade Federal da Paraíba. areia-PB. Brasil. https://repositorio.ufpb.br/jspui/bitstream/123456789/15363/1/DV018.pdf.

[35] Faria P.P., Cruz L.C.F., Sampaio S.A., Borges K.F., Minafra C.S. (2021). Biochemical analysis for broiler chicken-review. Nutr. Rev. Eletronica.

[36] Lee S.A., Nagalakshmi D., Raju M.V.L.N., Rao S.V.R., Bedford M.R., Walk C.L. (2019). Phytase as an alleviator of high-temperature stress in broilers fed adequate and low dietary calcium. Poult. Sci..

[37] Zhang Y.N., Wang S., Li K.C., Ruan D., Chen W., Xia W.G., Wang S.L., Abouelezz K.F.M., Zheng C.T. (2020). Estimation of dietary zinc requirement for laying duck breeders: Effects on productive and reproductive performance. egg quality. tibial characteristics. plasma biochemical and antioxidant indices. and zinc deposition. Poult. Sci..

[38] Lelis G.R., Albino L.F.T., Silva C.R., Rostagno H.S., Gomes P.C., Borsatto C.G. (2010). Phytase dietetic supplementation on nutrients metabolism of broilers. Rev. Bras. Zootec..

[39] Baldissera M.D., Baldisserotto B. (2023). Creatine Kinase Activity as an Indicator of Energetic Impairment and Tissue Damage in Fish: A Review. Fishes.

[40] Kong F., Zhao G., He Z., Sun J., Wang X., Liu D., Zhu D., Liu R., Wen J. (2021). Serum Creatine Kinase as a Biomarker to Predict Wooden Breast in vivo for Chicken Breeding. Front. Physiol..

[41] Barbosa A.A., Müller E.S., Moraes G.H.K., Umigi R.T., Barreto S.L.T., Ferreira R.M. (2010). Perfil da aspartato aminotransferase e alanina aminotransferase e biometria do fígado de codornas japonesas. Rev. Bras. Zootec..

